# A Qualitative Study of the Knowledge of Metabolic Syndrome, Attitudes about Lifestyle Modifications, and Preferences for Lifestyle Interventions among Patients with Cancer and Metabolic Syndrome

**DOI:** 10.3390/cancers16183147

**Published:** 2024-09-13

**Authors:** Isabel Martinez Leal, Ashwathy B. Pillai, Jessica T. Foreman, Kimberly W. Siu, Natalia I. Heredia, Carmen P. Escalante, Ellen F. Manzullo, Aimee J. Christie, Tamara E. Lacourt, Zayd Adnan Razouki, Jessica P. Hwang

**Affiliations:** 1 Department of Behavioral Science, The University of Texas MD Anderson Cancer Center, Houston, TX 77030, USA; 2 General Internal Medicine Department, The University of Texas MD Anderson Cancer Center, Houston, TX 77030, USA; abalachandran1@mdanderson.org (A.B.P.); jtristan@mdanderson.org (J.T.F.); cescalan@mdanderson.org (C.P.E.); emanzull@mdanderson.org (E.F.M.); zarazouki@mdanderson.org (Z.A.R.); jphwang@mdanderson.org (J.P.H.); 3 School of Public Health, The University of Washington, Seattle, WA 98195, USA; kimberlysiu87@gmail.com; 4 Department of Symptom Research, The University of Texas MD Anderson Cancer Center, Houston, TX 77030, USA; natalia.i.heredia@uth.tmc.edu; 5 Department of Health Promotion and Behavioral Sciences, The University of Texas Health Science Center at Houston, Houston, TX 77030, USA; ajchristie@mdanderson.org; 6 Department of Palliative, Rehabilitation and Integrative Medicine, The University of Texas MD Anderson Cancer Center, Houston, TX 77030, USA; tlacourt@mdanderson.org

**Keywords:** metabolic syndrome, cancer, healthy lifestyle, qualitative research

## Abstract

**Simple Summary:**

Metabolic syndrome is a combination of conditions that together increase one’s risk of heart disease, stroke, and diabetes and of developing colon, liver, and breast cancers. Metabolic syndrome is treatable using lifestyle changes and/or medication. However, clear, professional guidelines are lacking to direct physicians on how to best manage this condition among cancer patients. Consistent with patient-centered care, we interviewed cancer patients with metabolic syndrome on their knowledge, attitudes, and preferences regarding its management. The findings indicate that patients (1) were unfamiliar with metabolic syndrome, (2) preferred making lifestyle changes to taking medications to manage it, (3) identified family as a support and cancer-related treatment side effects as a barrier to exercising and managing this condition, (4) wanted clear communication with their medical team, and (5) wanted to share in developing personalized care plans with their team. This research contributes to the development of metabolic syndrome interventions tailored to cancer patients’ needs and preferences.

**Abstract:**

Metabolic syndrome is a group of disorders—hypertension, dyslipidemia, obesity, and insulin resistance—that together increase the risk of coronary heart disease, stroke, and diabetes. Although ~60% of cancer patients have metabolic syndrome, which increases their risk of mortality, oncology providers lack clear guidance about its management. The development of metabolic syndrome lifestyle interventions requires a better understanding of these patients’ knowledge, attitudes, and intervention preferences in order to inform management. During 2022–2023, 19 adult cancer patients with metabolic syndrome engaged in semistructured interviews focused on metabolic syndrome and lifestyle interventions. Interviews were analyzed using hybrid thematic analysis involving deductive and inductive coding. The findings indicated that patients lack knowledge about metabolic syndrome, were motivated to prioritize lifestyle modifications, and expressed strong interest in personalized care plans focused on healthy lifestyle rather than simply on weight loss. As part of their tailored intervention plans, participants desired clear communication with, and coordination of care among, their medical team and shared decision-making with providers about treatment decisions. The findings indicate that patients with metabolic syndrome want collaborative, patient-centered care, tailored interventions, and practical implementation strategies. This research will be used to inform the development of future lifestyle interventions for patients diagnosed with metabolic syndrome based on their identified needs and preferences.

## 1. Introduction

Metabolic syndrome is characterized by hypertension, dyslipidemia, insulin resistance, and obesity and is defined by the presence of at least three of the following five components: elevated waist circumference, elevated triglyceride levels, reduced high-density lipoprotein cholesterol level, elevated blood pressure, and elevated fasting glucose level [1]. Metabolic syndrome affects nearly 35% of United States (US) adults, and the prevalence increases with age, such that metabolic syndrome affects nearly 50% of individuals 60 years and older [2], a group at heightened risk for cancer [3]. Patients with cancer and metabolic syndrome have poor clinical outcomes. Metabolic syndrome can affect the tumor microenvironment through adipose tissue inflammation and lead to tumor growth [4]. Obesity has been associated with altered levels of insulin, insulin-like growth factor-1, steroid hormones, cytokines, leptin, and adiponectin [5]. A large, population-based study of 14,916 patients demonstrated that metabolic syndrome was associated with a 33% elevated total cancer mortality for patients compared to those without this metabolic condition [6].

Previous research indicates that metabolic syndrome is associated with an increased risk of cancer-specific mortality among patients with colon cancer (relative risk 1.72, 95% CI 1.03–2.42) [7], gastrointestinal cancer (esophageal, gastric, or colorectal) (HR 1.64, 95% CI 1.18–2.28) [8], breast cancer (HR 1.73, 95% CI 1.09–2.75) [9], endometrial cancer (HR 1.28, 95% CI 1.09–1.53) [10], and urothelial cancer (HR 1.38, 95% CI 1.01–1.89) [11]. Metabolic syndrome has also been associated with increased risk of recurrence among patients with colon cancer (HR 2.11, 95% CI 1.23–2.81) [12] and with progression from castration-sensitive to castration-resistant prostate cancer (HR 1.41, 95% CI 1.09–1.81) [13]. Additionally, cancer treatments can lead to alterations in insulin sensitivity, endothelial damage, lipid metabolism, hormone deficiencies, and obesity, all metabolic dysfunctions that contribute to developing metabolic syndrome [14].

Lifestyle interventions are recommended as effective strategies to control and treat metabolic syndrome among cancer patients, including those with cancer-treatment-induced metabolic syndrome [15]. Both cancer and metabolic syndrome are chronic conditions that have proven amenable to lifestyle interventions that target shared modifiable risk factors, i.e., obesity, unhealthy diet, physical inactivity, alcohol, and smoking [16]. As such, oncology providers have a potential interventional role to control the progression of metabolic syndrome among cancer patients to improve their outcomes.

Despite the growing body of evidence showing the harmful impact of metabolic syndrome on cancer development and clinical outcomes, there is no clear guidance for oncology providers regarding the management of metabolic syndrome. National oncology recommendations have focused on obesity and weight loss [17,18,19]. Oncology providers often do not ask patients about their diet [20], so it is unclear what patients know about metabolic conditions and what patients with metabolic syndrome need from their medical team to learn how to manage this syndrome. To be feasible, effective, and of high-quality, any intervention into managing metabolic syndrome for cancer patients should be informed by their preferences, needs, and values [21]. The aim of this preliminary research is to fill the gap in the existing literature on cancer patients’ needs, perspectives, and intervention preferences on treating metabolic syndrome. Given its exploratory nature, a qualitative study was selected as being best suited to capture the perspectives and experiences of cancer patients with metabolic syndrome regarding management of this condition and for exploring a research topic not previously examined [22,23]. In this qualitative study, we interviewed patients with cancer and metabolic syndrome to understand their knowledge of metabolic syndrome, attitudes about lifestyle modifications, and preferences for lifestyle interventions. The results of this preliminary study can ultimately be used to inform the development of future patient-centered care interventions.

## 2. Materials and Methods

### 2.1. Study Design and Ethical Approvals

The current work reports the findings of a qualitative study. An interview-based, exploratory qualitative research design was selected as little is known about cancer patients’ experiences and perspectives of metabolic syndrome. Qualitative research is recognized as best suited to capturing the meanings that people attribute to their experiences of illness, healthcare, treatment, and patient–provider interactions, and the complex relationships that pertain between these factors, which is critical to informing the quality of patient-centered care [24]. As a preliminary study, our focus was on understanding and responding to the lifestyle modification needs and preferences of cancer patients diagnosed with metabolic syndrome. This study adopted a social constructionist perspective [25], focused on how patients’ perceptions, knowledge, and experiences of metabolic syndrome and cancer were shaped and constructed via their social and medical relationships and interactions. The findings from this study will be used to inform the development of future lifestyle interventions to guide physicians and patients with cancer on managing metabolic syndrome. This study was reviewed and approved by the Institutional Review Board of The University of Texas MD Anderson Cancer Center prior to study commencement. In reporting this study, the authors followed the guidelines of the Standards for Reporting Qualitative Research (SRQR) Checklist (see Supplementary Table S1) [26].

### 2.2. Sampling Strategy

The target sample for this study was 20 participants. In the current study, our specific qualitative research questions guided the sample size and sampling strategy, data collection methods, and data analysis methods [25,27]. As this was a well-defined and structured study that addressed specific research questions using an interview format in which participants were all asked the same questions, we anticipated that saturation would be reached at 20 participants or fewer [28]. Saturation is the point at which data analysis no longer yields any new information or themes, producing only redundant findings [29].

### 2.3. Participants and Recruitment

Working closely with providers and clinic staff, the research team used purposeful sampling [22] to recruit participants between June 2022 and February 2023 from the MD Anderson General Internal Medicine Clinic. As metabolic syndrome affects individuals with various types of cancer, patients with a broad array of cancers and at different stages of treatment were purposefully recruited to gain a more comprehensive understanding of their experiences and preferences regarding management of metabolic syndrome. Additionally, as a preliminary study, we sought to capture the responses of a heterogenous group of patients living with cancer and metabolic syndrome to better understand their common needs and perspectives on managing this syndrome across their diversity. To be eligible, individuals had to be ≥18 years old, have a history of cancer, have been diagnosed with metabolic syndrome by a General Internal Medicine provider, and speak English. Potential participants were recruited through various means, including emails sent through the electronic medical record, phone calls, and provider and staff notification about the study. Individuals who expressed interest in participating were screened for eligibility by members of the research team. To maintain privacy, potential participants were contacted using secure messaging through the electronic medical record and using the Skype teleconferencing platform. Participants provided written consent through the electronic medical record prior to their engagement in the research. Each participant received a USD 25 gift card after completion of the interview and other protocol requirements, including a brief demographic survey.

### 2.4. Data Collection

A semistructured interview guide (see Supplementary Table S2) was developed based on a literature review as well as the research team’s expertise with treating metabolic syndrome among cancer patients and through conducting qualitative research. Three team members in the Department of General Internal Medicine—a clinical research fellow (A.B.P.), a program manager (J.T.F.), and a research coordinator (K.W.S.)—conducted individual interviews using the semistructured interview guide, with participants using the Zoom videoconferencing platform. Interviews generally lasted approximately 60 min. Prior to starting the interviews, interviewers discussed the nature of the study and the scope of the interview questions with participants. Team members conducting the interviews had no prior relationship with study participants before engaging them in the interview process. Interviews were audio-recorded and were later transcribed verbatim by a professional transcription service. To safeguard participants’ privacy, transcripts were de-identified and stored in a password-protected database along with audio-recordings. The questions focused specifically on discerning patients’ understanding of and attitudes towards metabolic syndrome and how this condition can affect cancer outcomes, what patients needed from their medical team to learn how to manage their metabolic syndrome, patient-identified facilitators of and barriers to adopting behavioral change, and preferences for future interventions. Demographic information was collected through a survey and from the electronic medical record.

### 2.5. Data Analysis

A hybrid approach to thematic analysis was used, involving deductive and inductive coding. This is a flexible analytic approach that entails an ongoing and iterative process of analysis based on the constant comparison method [30]. Initially, deductive coding was used. A priori or predetermined codes derived from the research aims, relevant literature, and the interview questions were used to develop a codebook consisting of codes with their definitions and criteria for use [31]. Transcripts were coded using the predetermined codes by a team of multidisciplinary researchers with expertise in general internal medicine (J.P.H., A.B.P.) and cultural anthropology and public health (I.M.L.). To limit researcher bias, all transcripts were divided between the 3 main analysts (J.P.H., A.B.P., I.M.L.), and each transcript was independently coded by at least 2 analysts. An iterative process was used for coding transcripts. The research team met weekly for 2 h over the span of ~4 months to review codes, note any emerging codes, and discuss coding until agreement was reached on the codebook. After team coding and review of the initial 10 transcripts, analysts began inductive coding to refine existing codes and develop new codes and categories drawn directly from the data. Moving to inductive coding during the latter stages of analysis supported an iterative, deeper dialogue with the data, to more fully understand patients’ perspectives [32]. This inductive coding process allowed us to discover meaning and patterns in the data to generate a posteriori, or empirical, codes drawn directly from the data. Team coding continued through a systematic process of synthesizing the data, in which codes were combined to form categories, and the relationships between categories were discerned and further abstracted to generate themes. K.W.S. assisted with final coding, analysis, and importing the dataset into Dedoose (version 9.0.90, Los Angeles, CA, USA: Sociocultural Research Consultants, LLC) to organize the data. Constant comparison was used to refine codes, confirm definitions, avoid redundancy, ensure accurate accounting of all the data, and substantiate attainment of saturation [33]. Analytic rigor was ensured through a process including researcher triangulation, reviewing and refining of codes through team coding, accurate accounting of all the data, and a final team discussion to ensure the analysis fit the data. The various analytic techniques used by the research team to ensure the rigor of the findings were in accordance with Guba’s seminal work [34] on the evaluative criteria for assessing the trustworthiness of qualitative research, through establishing transferability, or external validity; dependability, or reliability; credibility, or internal validity; and confirmability, or objectivity.

## 3. Results

### 3.1. Participants

Nineteen individuals participated in the study, most of them female and non-Hispanic White (Table 1). Thematic saturation was reached with these 19 participants, who expressed consistency across their responses [35]. Attainment of thematic saturation was confirmed through the failure of additional data analysis to generate any new information or themes, rendering continued interviewing redundant.

### 3.2. Qualitative Findings

Thematic analysis yielded five major themes: (1) understanding metabolic syndrome; (2) attitudes and approaches to managing metabolic syndrome; (3) capacity and limitations in managing metabolic syndrome; (4) patient-led care; and (5) tailored intervention plans. The themes focus on patients’ experiences and care preferences regarding metabolic syndrome, as well as provider recommendations. Table 2 shows themes and associated categories along with example quotes. Pseudonyms are used for the sources of the direct quotations to protect patient privacy.

#### 3.2.1. Theme 1: Understanding Metabolic Syndrome

When asked about their understanding of metabolic syndrome, patients gave responses indicating (1) *unfamiliarity with metabolic syndrome*, (2) *desire for education about metabolic syndrome and how it relates to cancer*, and (3) *lack of concern about metabolic syndrome*. Almost all patients reported being unfamiliar with metabolic syndrome prior to being diagnosed with this condition. While patients were familiar with the different risk factors that together constitute metabolic syndrome (i.e., hypertension, dyslipidemia, insulin resistance, and obesity), they were uninformed about this syndrome. Even those who were medically knowledgeable were unaware of metabolic syndrome. For example, one patient said, “I think a lot of it is education. I’ll be real honest with you. I’ve not really heard a lot about metabolic syndrome. These four criteria that you have here on the screen, obviously I’ve heard of since nursing school—but I didn’t really put them all together for this syndrome. So, when you’re talking to people about it, I don’t know that a lot of people are gonna really know what you mean when you say, ‘metabolic syndrome’” (Sam, 44 years old, central nervous system cancer). Most patients requested additional education on metabolic syndrome and its impact on cancer outcomes, recognizing the importance of this knowledge to effectively caring for themselves through lifestyle modifications. A few patients were unconcerned about their metabolic syndrome and either believed it was being controlled through medication or were concerned more about their cancer diagnosis than about their metabolic conditions. For example, in the following quote this patient expressed being unconcerned about making any changes to manage his metabolic syndrome, even though he was aware of being overweight: “I don’t really have any concerns because I’m just being honest with you. I mean, all three of those, the blood sugar, the blood pressure, and the cholesterol are well-controlled at the moment, so I hadn’t really had many concerns about it because the medication is controlling it. The weight is another thing. I am kind of heavy [laughter]. I mean, I’m just being honest with you… I’ve thought about it [exercising], but I’ve never put a lot of thought into it. I’m just gonna be quite honest with you [laughter]. I’ve thought, hey, I need to go walk another long time today or something, but I just don’t. I really don’t think a whole lot about it. I really don’t or haven’t.” (Robin, 59 years old colorectal cancer).

#### 3.2.2. Theme 2: Attitudes and Approaches to Managing Metabolic Syndrome

Patients’ attitudes about managing metabolic syndrome were centered on making lifestyle changes and included the following: (1) *lifestyle change is a priority*; (2) *motivation to change lifestyle: diagnosis as wake-up call and self-actualization by actively leading their lifestyle change*; and (3) *medications as adjuncts to lifestyle change*. Patients were clear that the means to address their metabolic conditions was to adopt lifestyle modifications to improve their long-term health. This attitude was based on patients’ views and experiences that, without lifestyle modifications, any other approaches to address underlying metabolic conditions, for example, weight gain through bariatric surgery or medication use only, would only result in short-term gains. Lifestyle modifications were necessary to sustain long-term changes. For example, one participant said: “They [providers] didn’t care how it was focusing on the scale number, versus really telling you how you could be the healthiest…The outcome or what the goal should be is feeling better. Feeling healthier. Being healthier. It’s not a matter of losing weight or a number on the scale. It’s a matter of feeling well and maintaining that healthy lifestyle…On the scale, I still weigh 142, and I was thinking, ‘That’s horrible after all this.’ Then it was one of the nutritionists that said, ‘Actually, that is not bad. You’re eating healthy and everything.’ See, if I’m just going with those numbers, that would’ve been one thing, but she was there to say, ‘Oh, no, you’re within that certain amount. The goal is you need to be healthy’” (Ryan, 67 years old, gynecologic cancer).

Many patients stated that receiving a diagnosis of diabetes or cancer served as what they termed a wake-up call, to motivate them to change their lifestyle. These patients understood the importance of behavioral changes to address risk factors contributing to metabolic syndrome and cancer, ultimately improving prognosis. Others expressed that their experience of being diagnosed with cancer motivated them to embark upon substantial lifestyle change and self-actualization to reach their potential. In fact, many patients preferred not to take medications to treat metabolic syndrome, except as an adjunct to behavioral changes. Several even stated that taking medications for metabolic syndrome motivated them to make lifestyle changes to reduce their reliance on medications, as in this example: “*The fact that I know that I don’t like to take medicine, and them adding another medication was like, okay, that’s it. That made it really easy. I don’t want you guys to keep adding different medications for different reasons. The best thing to do is to lose weight*” (Dylan, 56 years old, head and neck cancer). Medications were seen as being necessary in the short term until symptoms and conditions improved, but patients described their desire to eventually eliminate medications by incorporating behavioral changes for long-term health.

#### 3.2.3. Theme 3: Capacity and Limitations in Managing Metabolic Syndrome

Understanding the factors that patients identified as either aiding or limiting the management of their metabolic conditions is an essential step in developing a patient-centered intervention plan. Patients identified both (1) *supports to making lifestyle changes*, including (a) *identifying eating strategies to support healthy eating* and (b) *family support for diet and exercise changes*, and (2) *challenges to making lifestyle changes*, including (a) *financial limitations*, (b) *cancer-related weight loss*, and (c) *cancer treatment side effects*. Patients were generally well informed about what constitutes a healthy diet and physical exercise program and what worked and did not work personally for them. Many had identified and established routines to encourage healthy behaviors, such as not eating past satiation, not keeping unhealthy snacks at home, meal planning, daily exercise schedules, and low-impact workouts. Family members could serve as either a primary barrier to or a facilitator of patients’ lifestyle changes, depending upon whether or not family members also adopted the diet or exercise program. One patient noted the value of having family members as motivating exercise buddies: “*It does help to have an exercise buddy.…My daughter joined the Y. Then my granddaughter, who’s in her early 20s, has also joined the Y.…I have somebody with me most of the time, I guess, that I’m at the Y. Which does help. It does help to have somebody else. Plus, when my daughter’s there , she’s like, “Faster, faster, faster*”” (Alex, 72 years old, gynecologic cancer).

Of particular concern for people with cancer who have also been diagnosed with metabolic syndrome are the interactions and side effects of cancer treatments that can exacerbate this condition. Many patients reported that their cancer treatments adversely affected their ability to manage metabolic syndrome, either directly or indirectly. A number stated that some of their cancer medications, such as steroids, caused their blood sugar levels to rise significantly, exacerbating their metabolic syndrome and necessitating additional treatments, which led to yet other side effects: “*After I have a treatment my blood sugar will easily go up to the 300 level because of the steroid. It normally would go up and then it’d come back down and has not been doing that recently for the last couple of years. The blood sugar’s been high to the point that my doctor wanted to put me on insulin. I absolutely, positively, do not want to get to the point where I need insulin… Something would come back. I’d find a tumor. They would hit it for several months. Then, of course my blood sugar goes up. They say it’s dangerous to have a high blood sugar like that, so they put you on metformin or one of the other drugs. Then that creates other problems and issues*” (Quinn, 74 years old, non-Hodgkin’s lymphoma). While other patients reported that chemotherapy resulted in well-known cancer-related side effects, such as peripheral neuropathy or fatigue, which effectively hindered them from managing their metabolic syndrome through exercising: “*Coming off of chemo. Unfortunately, the chemo left me with some neuropathy in my feet. I had to make a decision of if I wanted to be active and manage the pain and limit some of the neuropathy or not*” (Jodie, gynecological cancer, 66). Meanwhile, another patient addressed the limitations of treatment-related fatigue: “*Most times, the chemo would have me so sick, I didn’t have time to think about anything else but tryin’ to feel better… Years ago, when I had better health, I used to exercise daily, but rigorous treatments of chemo, that tears your body down. I have to take Ritalin, now, just for the fatigue. I don’t do a whole lot of exercising*” (Riley, 68 years old, lung cancer).

#### 3.2.4. Theme 4: Patient-Led Care

Patients described what they needed from their medical team to better manage their metabolic conditions as well as their preferences regarding treatment, which included the following: (1) *communication: clear, direct communication from medical providers* and *practical communication tools*; (2) *collaborative care: understanding and respecting patients’ autonomy, attitudes, needs, and preferences*; and (3) *coordination of care among members of the patient’s medical team.* Patients valued clear and direct communication with their team concerning their metabolic conditions. In addition, patients wanted a collaborative, team approach to their care, centered on patients’ needs, preferences, and autonomy. For example, one patient said, “*It’s been the team together that has helped me feel like I could ask the questions that I needed and get the education and support that I needed to do what I needed to do. That’s very good. Allowing the patient to have the resources that they need to take control. In other words, ultimately, the doctor can be there and prescribe medicine and everything, but the patient has to take control of making the decision on this. I’m gonna do this*” (Jodie, 66 years old, gynecologic cancer). Management of cancer patients with metabolic syndrome is complex and requires coordinating efforts and treatments among a diverse medical team. Patients reported that including them within the communication and coordination of these efforts was important to providing them with quality of care and reassurance: “*They were working together as a team. If I did have that question, they would get me hooked up with somebody that could help me to answer that question… In other words, it’s been a team together that has helped me feel like I could ask the questions that I needed and get the education and support that I needed to do what I needed to do. That was good. That’s very good*” (Sarah, 67 years old, gynecological cancer).

#### 3.2.5. Theme 5: Tailored Intervention Plans

Most patients adopted an active approach to managing their metabolic conditions, seeking partnerships with their medical team focused on the following: (1) *co-creation of tailored intervention plans*; (2) *offering instructions tailored to patients’ needs, resources, and support*; and (3) *regular monitoring, feedback, and plan adjustment*. Patients were aware that many individual differences could influence intervention plans, necessitating a flexible approach rather than a standardized treatment approach for each patient. Patients commonly reported needing practical and structured instructions on lifestyle modifications, including nutritional recipes, specific physical exercises, and what to order when eating out. Practical information and guides that could assist patients in their everyday management of metabolic syndrome were highly valued. Patients wanted to actively contribute as participants in their care: *I feel that when I go see my doctor that when I walk out, I should have a set of instructions that I go do. In other words, I don’t’ think it’s all “I’m gonna give you…, I’m gonna tell you what’s wrong, or I’m gonna tell you here, and I’m gonna give you this pill and you go home.” … I would like to see just like in school. We’re gonna teach you something. Here’s what you’re gonna learn. Now, you go home and practice this. Here’s what you need to be doing, again, to take your responsibility of this—fixing your health. It’s not my health, from the doctor’s standpoint, but it’s what I need to do on my end. You need these doctors to tell you what you need to do and give you an action plan because it’s not always just a pill you’ve gotta’ take to fix the problem.* (Quinn, 74 years old, non-Hodgkin’s lymphoma).

Particularly important to patients was the development of a structured plan that included regular monitoring and evaluation. Patients noted that monitoring could be done online using secure patient portals in order to facilitate the adjustment of plans as needed. For example, one patient said, “*I think that, because there’s different components to metabolic syndrome, I wanna’ know, ‘Where am I in that scale?’ ‘This is what your main—what the main concern is for metabolic syndrome for you. We’re going to target two things out of the different components of it’… So, having my team check in on me on a routine basis, I think, is important. Months will go by that I’m like, ‘How am I really doing? I need to check in with somebody.’ Having someone check in with me, and see where I’m at, and see what they can do to help me. I think it would be great, definitely*” (Parker, 58 years old, head and neck cancer).

## 4. Discussion

In this preliminary qualitative study of patients with cancer and metabolic syndrome, we found that patients had limited knowledge of metabolic syndrome and its consequences related to cancer. Most of the participants desired additional information and education, although a few expressed a focus on their cancer trajectory over metabolic health. In addition, many patients reported that their cancer or diabetes diagnosis served as a wake-up call, engendering resilience, and motivation to make lifestyle modifications a priority. Many patients were willing to take medications as an adjunct to achieving their larger goals. Importantly, patients expressed a strong interest in developing a personalized and holistic care plan focused on a healthy lifestyle rather than on simply achieving weight loss. Patients wanted a care plan based on their preferences and capabilities. Patients wanted clear bidirectional communication with their providers, opportunities for their providers to monitor progress, and coordination of care among members of their medical team.

Our findings corroborate the scant previous literature [36,37] on cancer patients’ understanding of metabolic syndrome and its relationship to cancer outcomes. Seo et al. [36] found that, while 56.8% of participants had heard of metabolic syndrome, their knowledge of the syndrome was poor, and 52.3% wanted further information from their medical provider about this condition. Similarly, Jang et al. [37] found that, while 70% of participants had heard of metabolic syndrome, their knowledge of the syndrome was poor, and 64.3% wanted further information from their medical provider about this condition. However, whereas Seo et al. [36] hypothesized that participants’ focus on their cancer progression rather than metabolic syndrome drove their lack of knowledge about this condition, our findings show the opposite, that only a few participants prioritized their cancer progression over metabolic syndrome.

It is crucial to understand patients’ informational needs regarding metabolic syndrome given the high prevalence of metabolic syndrome among cancer patients, which ranges from 24 to 60%, depending on cancer type [11,38,39], and the increased risk of cancer-specific mortality associated with metabolic syndrome [7,8,9,10,11]. Furthermore, common cancer treatments such as surgery, radiotherapy, chemotherapy, and hormonal therapy can induce metabolic syndrome [15], worsening patients’ long-term outcomes.

Informing patients about metabolic syndrome is essential in equipping them with the tools they need to manage this condition [40]. Thus, the development of education resources for patients is critical. In addition, given that existing national oncology recommendations have primarily focused on obesity and weight loss [17,18,19,20] and neglected the larger context of overall health promotion for patients, new evidence-based education and practice guidelines for providers that are informed by and focused on patient-centered health outcomes, such as the guidelines by Wharton et al. [41], will be key to addressing metabolic syndrome. A 2018 American Society of Clinical Oncology (ASCO) survey of the oncology workforce showed that providers may find it difficult to incorporate lifestyle modifications into patient treatment plans because of lack of education on these topics, lack of time, or lack of appropriate programs to which to refer patients [20]. The development of new education and practice guidelines will help providers overcome these barriers. The ASCO survey also showed that providers perceive resistance from patients to making lifestyle changes [20], which could make providers reluctant to initiate these discussions. However, the findings from our current study suggest that patients are open and ready to receive information and collaborate with providers to identify personalized lifestyle modifications.

Most patients identified lifestyle modifications as their preferred approach to managing metabolic syndrome, with medication use being supplementary if and when required. Many stated that receiving a cancer or diabetes diagnosis served as a wake-up call, spurring them to take charge of their health and implement a self-actualization plan, despite various challenges during their cancer trajectory. Patients adopted a committed attitude to persevering through challenges in order to develop flexible action plans that fit their capabilities. Recent literature aligns with our study findings, showing that cancer patients’ needs and challenges regarding diet and physical exercise vary over the course of their survivorship [42], signaling the importance of provider support throughout the cancer trajectory. In a population-based prospective cohort study of 1696 breast cancer survivors, exercise participation and duration increased from 6 months after diagnosis to 18 and 36 months after diagnosis and favored lower-impact activities such as walking [38]. The findings from other studies showed that the prevalence of metabolic syndrome at 5 years after cancer diagnosis was lower among survivors who participated in exercise for at least 30 min every day (OR 0.69, 95% CI 0.48–0.98) than among survivors who reported no exercise, supporting previous research on the benefits of physical exercise for those with metabolic syndrome [43,44,45]. A recent study demonstrated that a lifestyle intervention based on provider coaching benefited patients by increasing self-efficacy, goal setting, and self-monitoring of results [46]. Provider coaching was also associated with perceived increased family and provider social support to sustain behavioral changes [47]. These studies highlight the importance of collaborative relationships between providers and patients, which ultimately empower patients to make healthy lifestyle changes [48].

We found that patients wanted to engage with their medical team and participate in a personalized treatment plan for their metabolic health issues and lifestyle modifications. This would be in line with a previous call to action to use a patient-centered model within a disease–illness framework to manage obesity [49]. Patients requested clear communication and monitoring plans with their medical providers. Previous studies have shown that cancer patients who perceive a lack of support from healthcare providers may experience uncertainty in how to implement lifestyle modifications [50]. This indicates that patients with cancer would benefit from providers who employ open and direct communication styles. Patients also expressed a desire to collaborate with providers about treatment decisions. In one cluster-randomized clinical trial of shared decision-making in patients with diabetes, patients in the shared decision-making group had higher rates of achieving treatment targets for hemoglobin A1c, blood pressure, and total cholesterol after 24 months compared to patients in the usual care group [51]. Providers need support and guidance from oncology organizations to implement shared discussions and develop true collaboration to reach agreement about complicated health decisions [52].

### Limitations and Future Research

Given the likelihood of inherent biases of self-selection, our study results may not be applicable to cancer patients with metabolic syndrome who did not participate. Additionally, our study reflected the experiences of mostly female, non-Hispanic, White, English-speaking cancer patients with solid tumors. Future research should examine racially and ethnically diverse cancer patients’ experiences of managing metabolic syndrome. Our findings may not be applicable to patients who differ from our study population, such as those currently receiving anticancer therapy, who made up only ~26% of our sample. A primary aim underlying this research is that the findings of this preliminary study will inform the development and assessment of future lifestyle interventions for cancer patients with metabolic syndrome. For example, future quantitative research is warranted on assessing the effect of appropriately matched therapeutic lifestyle medicine approaches to patients on adjuvant treatment after radical anticancer therapy. The role of metabolic syndrome is unknown and may be marginal in patients receiving palliative therapy; thus, therapeutic lifestyle modifications may not be suitable for this group.

We believe that our study’s strength lies in its focus on patient experiences as described by a diverse group of patients themselves [27,53,54]. The commonality of themes expressed across our heterogeneous sample supports a greater generalizability of findings than is possible in a more homogenous group, thus extending the applicability, transferability, and relevance of the findings of this preliminary study in informing future lifestyle interventions for these patients. Learning about patients’ perspective allows providers to understand patients’ knowledge and gain from their wisdom and advice [55]. Patients’ consistent responses and emphasis on patient-centered and personalized approaches to managing metabolic syndrome provide us with valuable information for updating clinical guidelines. Methodologically, the use of a hybrid, inductive/deductive thematic analysis is another strength of this study. Prior research has demonstrated the validity of hybrid, inductive/deductive thematic analysis [56]. Combining these two approaches serves to overcome the particular weakness of each, while supporting their respective analytic strengths. Inductive methods are faulted for being more susceptible to researcher bias but also are prized as data-driven and capable of generating new ideas and theories. Deductive methods that use predetermined codes can fail to capture important information shared by participants but also are usually developed through consultation of extant literature and knowledge and thus are less prone to bias. In this study, various methods were used to safeguard against the potential for researcher bias. For example, each transcript was independently double- or triple-coded and was then reviewed and refined through team coding. Lastly, the research team discussed and developed findings throughout the analytic process to confirm that data supported the final interpretation. Our approach of starting with deductive coding and then moving on to inductive coding allowed for the generation of themes that were ultimately driven by the data and grounded in patients’ lived experiences. The integration of inductive and deductive methods yielded a more balanced and comprehensive understanding of the data [57].

## 5. Conclusions

To our knowledge, this is the first qualitative study conducted in the US aimed at contributing patients’ knowledge and perspectives of metabolic syndrome, and attitudes towards lifestyle modifications and preferences, to the development of patient-centered care interventions. Our preliminary study findings have relevance for oncology providers treating patients with cancer and metabolic syndrome at any point in the cancer journey. Improving the metabolic health of patients with cancer has a direct impact on their cancer-specific survival [7,8,9,10,11,58]. Our study findings may also be useful to primary care providers who care for patients with metabolic syndrome after their active cancer treatment; such providers may be well positioned to offer guidance about lifestyle modifications.

In summary, our study findings showed that patients with metabolic syndrome and cancer need a patient-centered and personalized care plan. Future work is needed to design educational interventions regarding metabolic syndrome for patients with cancer. This study also contributed towards, and highlighted the necessity of, updating clinical guidelines for providers that are informed by patients’ perspectives and preferences. A collaborative team approach among oncologists, primary cancer physicians, advanced practice providers, dieticians, and others on the multidisciplinary team is essential in order to offer comprehensive therapeutic lifestyle interventions. Collaborating with patients to identify which lifestyle modifications they would be interested in pursuing is essential to creating a personalized approach. Implementation of practical management and monitoring strategies for patients with cancer and metabolic syndrome, along with longitudinal assessment of their metabolic endpoints, will be critical to assess the impact of interventions.

## Figures and Tables

**Table 1:** Participant characteristics (N = 19) ^a^.

Characteristic	Value
Age, mean (SD), y	64.6 (13.4)
Gender	
Female	12 (63)
Male	7 (37)
Race	
Asian	2 (11)
Black	3 (16)
White	12 (63)
Two or more races	2 (11)
Ethnicity	
Hispanic	3 (16)
Non-Hispanic	16 (84)
Born in the US	
Yes	16 (84)
Marital status	
Married	15 (79)
Separated	2 (11)
Divorced	1 (5)
Widowed	1 (5)
Education	
High school diploma	8 (42)
Bachelor’s degree	6 (32)
Master’s degree	2 (11)
Doctoral degree	2 (11)
Professional degree	1 (5)
Primary cancer diagnosis	
Gynecologic	5 (26)
Genitourinary	4 (21)
Colorectal	3 (16)
Breast	2 (11)
Head & neck	2 (11)
Central nervous system	1 (5)
Lung	1 (5)
Non-Hodgkin lymphoma	1 (5)
Systemic cancer treatment	
Actively receiving	5 (26)
Completed	14 (74)

^a^ Values in the table are number of patients (percentage) unless otherwise indicated.

**Table 2:** Themes, associated categories, and selected exemplary quotes.

Themes		Categories		Selected Exemplary Quotes
Knowledge of metabolic syndrome	Understanding metabolic syndrome	Unfamiliarity with metabolic syndrome		“[Metabolic syndrome] was never fully explained to me…this is what this is, and this is what you can do to control it” (Parker, 58 years old, head & neck cancer).
		Desire for education about metabolic syndrome and how it relates to cancer		“There’s not a lot of education on exactly what is the best for you. I think that was a big game changer for me. I had a desire to, first of all, find out—Okay, especially not only with the metabolic syndrome but with my cancer and everything. I found out that a lotta this is connected. What was the best thing for me to do nutrition-wise? That really helped me out. I think education would be the number one thing” (Ryan, 67 years old, gynecologic cancer).
		Lack of concern about metabolic syndrome		“Let me put this in context. I’m really not worried about metabolic syndrome; my chief concern is treating the cancer. Something’s going to kill me. I’m 84 years old, and this is an end game” (Taylor, 84 years old, genitourinary cancer).
Attitudes about lifestyle modifications	Attitudes and approaches to managing metabolic syndrome	Lifestyle change is priority		“I think the most important thing is the lifestyle adjustment” (Jodie, 66 years old, gynecologic cancer).
		Motivation to change lifestyle	Diagnosis as wake-up call	“For me, cancer was a really big wake-up call. When you’re faced with life and death you see things differently…it scares the bejabbers out of you. In my mind, I didn’t have a choice. It was either do that [change lifestyle] or I risk the chance of dying and I’ve got four kids at home. I’m not willing to risk my chance of dying” (Drew, 52 years old, gynecologic cancer).
			Self-actualization by actively leading lifestyle change	“It was a personal commitment that I wanted to make a difference in my own lifestyle—what is it that I wanna’ do in my life? Once, if that is clearly defined, then you put in action plans which are needed to meet that goal.…I don’t think any limitations have any impact on it.…That’s what you have to do. Once you make the commitment you stick to it” (Jessie, 83 years old, genitourinary cancer).
		Medications as adjuncts to lifestyle change		“Anything that I can do to help eliminate [medications] is a huge plus in my book.…I think the most important thing is you try to change your eating habits and exercise.…I never thought that those were gonna be truly important in your life, but it is. It’s made a huge difference in my life…if I end up doing all that and trying to fix it on my own and I still can’t, I have to understand that it’s hereditary and it’s [medication] part of what I have to do for the rest of my life.…It’s important to have some kind of control that you can control the situation a bit by eating better and exercising, I think it goes a long way. I’m a true believer in that” (Parker, 58 years old, head & neck cancer).
	Capacity and limitations in managing metabolic syndrome	Supports to making lifestyle changes	Identifying eating strategies to support heathy eating	“Now, we live in an area with several people all around us that are very social, and there’s always events going on. I guess that’s one of the biggest barriers, is that you’re not—unless you physically bring the right food all the time, it’s not always available. You either have to eat before you go or after you come back and that type of thing” (Jodie, 66 years old, gynecologic cancer).
			Family support for diet and exercise changes	“Exercise, adding the exercise, tryin’ to get mobility, and the military has given me some exercises that I can do that’s low impact on my knees and my hips.…My mom, she went from a size 28 to a 6, 8 now.…A big part of what she does daily is exercise.…She’s very encouraging” (Dylan, 56 years old, head & neck cancer).
		Challenges to making lifestyle changes	Financial limitations	“Right now, I would say it’s more economics, like everything’s getting more expensive. Right now, it’s like, you cannot really pick what you want, like organic this or organic that, so that would be a challenge” (Kai, 57 years old, gynecologic cancer).
			Cancer-related weight loss	“My problem, now, is not that—I’m never hungry. Most of the times, I make myself eat because I take medication, and bein’ a cancer patient, I need to keep weight on. I try to eat even when I really don’t want to. The food tastes—still tastes good. It’s just my appetite. I really don’t want it. I don’t have a weight problem. I have a problem with keepin’ the weight I have on” (Riley, 68 years old, lung cancer).
			Cancer treatment side effects	“Mobility is a little hard because the enfortumab really did produce a significant peripheral neuropathy both afferent and efferent. I did a lot of falling early on” (Taylor, 84 years old, genitourinary cancer).
Preferences for lifestyle interventions	Patient-led care	Communication	Clear, direct communication from medical providers	“I’m all about people just being straightforward and honest and saying, ’Here’s the issue, and here’s what we need to do to help fix it,’ and so just kinda straightforward, shoot it to me straight, and tell me what I can do to make it better and—probably works best for me” (Kai, 57 years old, gynecologic cancer).
			Practical communication tools	“I really like the MyChart because that gives me the ability to communicate with the nurses and provide that feedback and get feedback from them” (Quinn, 74 years old, non-Hodgkin lymphoma).
		Collaborative care	Understanding and respecting patients’ autonomy, attitudes, needs, and preferences	“When I was first in the hospital, and I was diagnosed with diabetes, because they diagnosed me when they found out I had cancer, they went straight to insulin versus trying to give me time to get on pill form, and I had an issue with that. ‘Cause I just didn’t wanna’ jump right into insulin. But when they let me go home and I told them I didn’t wanna’ be on insulin, I wanted to be on something pill form, and they did it” (Drew, 52 years old, gynecologic cancer).
		Coordination of care	Among members of the patient’s medical team	“The team was working together, I guess would be a good way to say. Team partnership on your whole health. I was seen by the—started out with my cancer doctors. Then they referred me to the cardiologist, the endocrinologist, and then to Dr. X, who also then got me into integrated medicine” (Ryan, 67 years old, gynecologic cancer).
	Co-creation of tailored intervention plans	Offering instructions tailored to patients’ needs, resources, and support		“An achievable goal for where I am in my health because not everybody can do the same. Not everybody’s in the same process of healing. Individualized. That’s what we’re coming to. Again, an individualized plan or goal for you where you are may not be the same as for Mr. X over here, who has the same, maybe, diagnosis but is in a different place” (Alex, 72 years old, gynecologic cancer).
		Regular monitoring, feedback, and plan adjustment		“It’s like I take the blood pressure measurement—the blood level—sugar level—every morning. Then I send that periodically to Dr. Y so that she can see the trend.…I try to do it once a month or whatever. It’s my way of giving her feedback that what she’s asked me to do, or what I needed to do, is working. It doesn’t require her to reply back. It’s just so that she can see that we’re going in the right direction.…That shows me that it’s working. If I didn’t see something that showed numbers that show that what you’re doing is making a difference, then I probably wouldn’t be doing it. It’s the feedback to me that keeps me going” (Quinn, 74 years old, non-Hodgkin lymphoma).

Note: Shading has been added to the table to facilitate differentiation of themes and ease of reading.

## Data Availability

Data are not publicly available due to privacy restrictions. The data that support the findings of this study are available from the senior author, J.P.H., upon reasonable request.

## Supplementary Materials

The following supporting information can be downloaded at https://www.mdpi.com/article/10.3390/cancers16183147/s1, Table S1: SRQR (Standards for Reporting Qualitative Research) Checklist. Table S2: Interview Guide. Reference [26] is cited in the supplementary materials.
