# Supplementary material for: A Qualitative Study of the Knowledge of Metabolic Syndrome, Attitudes about Lifestyle Modifications, and Preferences for Lifestyle Interventions among Patients with Cancer and Metabolic Syndrome
Source: Cancers (Basel). 2024 Sep 13;16(18):3147. doi: 10.3390/cancers16183147 (PMC11430367; doi:10.3390/cancers16183147)
Supplement: Supplementary file 1 [file cancers-16-03147-s001.zip › cancers-3169448-supplementary.pdf]

Supplementary Table S1: Standards for Reporting Qualitative Research (SRQR)

O'Brienk B.C.; Harris, I.B.; Beckman, T.J.; Reed, D.A.; Cook, D.A. Standards for reporting qualitative research: A synthesis of recommendations. *Acad. Med.* 2014*, 89*, 1245–1251. Available online: http://www.equator-network.org/reporting-guidelines/srqr/.

No. Topic	Item	Page number
**Title and abstract**		
S1 Title	Concise description of the nature and topic of the study identifying the study as qualitative or indicating the approach (e.g., ethnography, grounded theory) or data collection methods (e.g., interview, focus group) is recommended	1
S2 Abstract	Summary of key elements of the study using the abstract format of the intended publication; typically includes objective, methods, results, and conclusions	1
**Introduction**		
S3 Problem formulation	Description and significance of the problem/phenomenon studied; review of relevant theory and empirical work; problem statement	2
S4 Purpose or research question	Purpose of the study and specific objectives or questions	2
**Methods**		
S5 Qualitative approach and research paradigm	Qualitative approach (e.g., ethnography, grounded theory, case study, phenomenology, narrative research) and guiding theory if appropriate; identifying the research paradigm (e.g., positivist, constructivist/interpretivist) is also recommended	3
S6 Researcher characteristics and reflexivity	Researchers' characteristics that may influence the research, including personal attributes, qualifications/experience, relationship with participants, assumptions, or presuppositions; potential or actual interaction between researchers' characteristics and the research questions, approach, methods, results, or transferability	4, Table S2
S7 Context	Setting/site and salient contextual factors; rationale [a]	3
S8 Sampling strategy	How and why research participants, documents, or events were selected; criteria for deciding when no further sampling was necessary (e.g., sampling saturation); rationale [a]	3-4
S9 Ethical issues pertaining to human subjects	Documentation of approval by an appropriate ethics review board and participant consent, or explanation for lack thereof; other confidentiality and data security issues	3-4, 13
S10 Data collection methods	Types of data collected; details of data collection procedures including (as appropriate) start and stop dates of data collection and analysis, iterative process, triangulation of sources/methods, and modification of procedures in response to evolving study findings; rationale [a]	3-4

S11 Data collection instruments and technologies	Description of instruments (e.g., interview guides, questionnaires) and devices (e.g., audio recorders) used for data collection; if/how the instrument(s) changed over the course of the study	3,Supplementary Table S2
S12 Units of study	Number and relevant characteristics of participants, documents, or events included in the study; level of participation (could be reported in results)	3-5
S13 Data processing	Methods for processing data prior to and during analysis, including transcription, data entry, data management and security, verification of data integrity, data coding, and anonymization/deidentification of excerpts	3-4
S14 Data analysis	Process by which inferences, themes, etc., were identified and developed, including researchers involved in data analysis; usually references a specific paradigm or approach; rationale [a]	3-4
S15 Techniques to enhance trustworthiness	Techniques to enhance trustworthiness and credibility of data analysis (e.g., member checking, audit trail, triangulation); rationale [a]	4
**Results/Findings**		
S16 Synthesis and interpretation	Main findings (e.g., interpretations, inferences, and themes); might include development of a theory or model, or integration with prior research or theory	5-7
S17 Links to empirical data	Evidence (e.g., quotes, field notes, text excerpts, photographs) to substantiate analytic findings	6-11
**Discussion**		
S18 Integration with prior work, implications, transferability, and contribution(s) to the field	Short summary of main findings; explanation of how findings and conclusions connect to, support, elaborate on, or challenge conclusions of earlier scholarship; discussion of scope of application/generalizability; identification of unique contribution(s) to scholarship in a discipline or field	11-12
S19 Limitations	Trustworthiness and limitations of findings	12-13
**Other**		
S20 Conflicts of interest	Potential sources of influence or perceived influence on study conduct and conclusions; how these were managed	14
S21 Funding	Sources of funding and other support; role of funders in data collection, interpretation, and reporting	13

[a] The rationale should briefly discuss the justification for choosing that theory, approach, method, or technique rather than other options available, the assumptions and limitations implicit in those choices, and how those choices influence study conclusions and transferability. As appropriate, the rationale for several items might be discussed together.

Supplementary Table S2: Interview guide

1) As someone who has received treatment for cancer and metabolic syndrome, what are your top 3 concerns?
2) Think about the conversations you have had with health care providers about metabolic syndrome.
    a) What worked well and what did you like?
    b) What did you not like about the ways providers talked with you about metabolic syndrome?
3) How would you like your doctor or clinical team to speak with you about metabolic syndrome management?
    a) How do you want to know about metabolic syndrome and/or lifestyle management? How would you like to receive this information?
4) Some patients with metabolic syndrome take medications for their diabetes, high blood pressure, cholesterol, or weight.
    a) What do you think about medications that are used for treating these conditions?
    b) How do you feel about medications that can help with weight loss?
5) Some patients with metabolic syndrome are encouraged to make adjustments to their lifestyle, like eating healthier or exercising more, to help manage their metabolic conditions.
    a) What do you think about lifestyle modifications for treating these conditions?

    Some patients take medications for their metabolic syndrome, others choose lifestyle interventions, and other patients may do some of both.
    b) What do you think are the most important things that help with metabolic syndrome?
    c) What about bariatric surgery?
6) Now can you tell me about a time when you made (or thought about making) a change to your physical activity?
    a) What kind of changes were they?
    b) What made you want to make (or think about making) that change?
    c) What made it/might have made it difficult to make that change? Do you have any physical limitations that might prevent you from making changes to physical activity?
    d) What made it/might have made it easy to make that change?
    e) In the future, if you were to make another change to your physical activity, what would make it easier?
    f) What would make it more difficult?
    g) What helps you to be physically active?
7) Can you tell me about a time when you made (or thought about making) a change to your eating? What kind of changes were they?
    a) What made you want to make (or think about making) that change?

b) What made it/might have made it difficult to make that change?
c) Do you have any physical limitations that might prevent you from making changes to eating?
d) What made it/might have made it easier to make that change?
e) In the future, if you were to make another change to your eating, what would make it easier?
f) What would make it more difficult?

8) Have you ever tried to use weight control practices such as fasting or severely restricting your calorie intake?
9) Have you ever engaged in binge eating, where you eat a lot of food in a short amount of time while feeling a sense of loss of control?
10) Have you ever engaged in purging, such as taking laxatives or vomiting or excessive exercise, to control your weight?
    a) What about taking diet pills or stimulants or prescription medicine for weight loss? If yes, can you tell us about your experience with this? Are you currently using any of these practices?
11) What helps you stay focused on your eating habits?
12) Have you ever heard of a practice called mindful eating?
    a) If yes: Can you tell me what you know about it?
    b) If no, explain: Mindful eating is about focusing on the food in front of you and the act of eating, trying to let go of distractions, and focusing on your 5 senses while eating. It's about eating slowly, chewing thoroughly, and paying attention to your body's hunger and fullness signals.
    How interested are you in learning how to practice mindful eating? Why?
13) Have you ever heard of a practice called intuitive eating?
    a) If yes: Can you tell me what you know about it?
    b) If no, explain: Intuitive eating is about trusting your body to make food choices that feel good for you, trying not to judge yourself on what you eat, how much you eat, or when you eat. It's sometimes considered an alternative to dieting. How interested are you in learning how to practice intuitive eating? Why?
14) If you were king or queen for a day (if resources were not an issue), how would the clinic team support you with the kinds of healthy lifestyle goals we discussed today to meet the needs of patients like you?
